# ERα-related chromothripsis enhances concordant gene transcription on chromosome 17q11.1-q24.1 in luminal breast cancer

**DOI:** 10.1186/s12920-020-0729-7

**Published:** 2020-05-14

**Authors:** Chun-Lin Lin, Xi Tan, Meizhen Chen, Meena Kusi, Chia-Nung Hung, Chih-Wei Chou, Ya-Ting Hsu, Chiou-Miin Wang, Nameer Kirma, Chun-Liang Chen, Ching-Hung Lin, Kate I. Lathrop, Richard Elledge, Virginia G. Kaklamani, Kohzoh Mitsuya, Tim H.-M. Huang

**Affiliations:** 1 Department of Molecular Medicine, University of Texas Health Science Center at San Antonio, 7703 Floyd Curl Drive, San Antonio, TX 78229 USA; 2 Department of Oncology, National Taiwan University Hospital, Taipei, Taiwan; 3 Department of Internal Medicine, National Taiwan University Hospital, Taipei, Taiwan; 4 Department of Medicine, Mays Cancer Center, University of Texas Health Science Center at San Antonio, San Antonio, TX USA

**Keywords:** Luminal subtype breast cancer, Chromosomal rearrangement, ERα, Chromothripsis, Concordant transcription, Druggable target, Nanopore sequencing

## Abstract

**Background:**

Chromothripsis is an event of genomic instability leading to complex chromosomal alterations in cancer. Frequent long-range chromatin interactions between transcription factors (TFs) and targets may promote extensive translocations and copy-number alterations in proximal contact regions through inappropriate DNA stitching. Although studies have proposed models to explain the initiation of chromothripsis, few discussed how TFs influence this process for tumor progression.

**Methods:**

This study focused on genomic alterations in amplification associated regions within chromosome 17. Inter−/intra-chromosomal rearrangements were analyzed using whole genome sequencing data of breast tumors in the Cancer Genome Atlas (TCGA) cohort. Common ERα binding sites were defined based on MCF-7, T47D, and MDA-MB-134 breast cancer cell lines using univariate K-means clustering methods. Nanopore sequencing technology was applied to validate frequent rearrangements detected between ATC loci on 17q23 and an ERα hub on 20q13. The efficacy of pharmacological inhibition of a potentially druggable target gene on 17q23 was evaluated using breast cancer cell lines and patient-derived circulating breast tumor cells.

**Results:**

There are five adjoining regions from 17q11.1 to 17q24.1 being hotspots of chromothripsis. Inter−/intra-chromosomal rearrangements of these regions occurred more frequently in ERα-positive tumors than in ERα-negative tumors. In addition, the locations of the rearrangements were often mapped within or close to dense ERα binding sites localized on these five 17q regions or other chromosomes. This chromothriptic event was linked to concordant upregulation of 96 loci that predominantly regulate cell-cycle machineries in advanced luminal tumors. Genome-editing analysis confirmed that an ERα hub localized on 20q13 coordinately regulates a subset of these loci localized on 17q23 through long-range chromosome interactions. One of these loci, *Tousled Like Kinase 2* (*TLK2*) known to participate in DNA damage checkpoint control, is an actionable target using phenothiazine antipsychotics (PTZs). The antiproliferative effect of PTZs was prominent in high TLK2-expressing cells, compared to low expressing cells.

**Conclusion:**

This study demonstrates a new approach for identifying tumorigenic drivers from genomic regions highly susceptible to ERα-related chromothripsis. We found a group of luminal breast tumors displaying 17q-related chromothripsis for which antipsychotics can be repurposed as treatment adjuncts.

## Background

Genomic alterations harboring tumor-promoting loci, including *ERBB2*, *FOXA1*, and *MET*, in primary tumors are frequently linked to aggressive phenotypes [1–3]. Neighboring loci mapped within the same chromosome locations may be co-regulated with these genes [2, 4, 5]. Less is known about *cis*- and *trans*-regulatory mechanisms of these co-regulated loci and their functional roles as tumorigenic drivers. We and other investigators found that estrogen stimulation triggers estrogen receptor α (ERα) to interact with target promoters and coordinately regulate transcription of multiple protein-coding genes through intra-chromatin looping in the neighborhood or inter-chromatin interactions in distant chromosome regions [6, 7]. Further studies indicated that topologically associating domains (TADs) respond to ligand stimulation as transcription units, within which gene activation or repression is concordantly regulated in response to this hormone signaling [6, 8]. Frequent inter−/intra-TAD interactions promote rearrangements at chromatin interacting sites due to erroneous DNA repair in unstable cancer genomes [9]. As a result, massive chromosome alterations can occur in these TADs leading to chromothripsis [6, 10]. In a sense, this chromothriptic event could be a “chromatin relic” from which prior proximity interactions promote aberrant exchanges between two chromosomes during tumorigenesis.

Chromothripsis, a complex genomic rearrangement due to chromosome shattering and aberrant stitching, has previously been reported as a hallmark of large-scale genome instability in breast cancer [11]. When chromothriptic alterations involve multiple chromosomes, fragments of different chromosomes are exchanged and joined as pairs [12, 13]. The genomic rearrangements can affect regulatory activity and expression of oncogenes and tumor suppressor genes, leading to malignant transformation [14]. A pan-cancer study, using whole genome data from the International Cancer Genome Consortium (ICGC) and the Cancer Genome Atlas (TCGA), indicated that chromothripsis is a frequent event during tumorigenesis and that affected chromosomes vary among different tumor subtypes [15]. Furthermore, chromosome 17 was observed to have the highest density of chromosome translocations and copy-number alterations in breast cancer [16], suggesting that this chromothriptic occurrence is not a stochastic event. Given that, this study aims to unravel how ERα-related chromothripsis increases concordant gene transcription on chromosome 17 and to provide preliminary evidence of an actionable target for luminal breast tumors carrying 17q23 amplification.

## Methods

### Datasets

In silico analysis was conducted using genomic and transcriptomic data of TCGA breast invasive carcinoma cohort downloaded from cBioPortal (http://download.cbioportal.org/brca_tcga.tar.gz). A total of 1014 primary tumor samples of ERα-positive and -negative types were available with both mRNA-seq gene expression and copy number data, and 96 adjacent normal tissue samples with gene expression data. Copy number values were assigned to defined 100-kb segments across the genome, and segmented copy number value 0 was regarded as diploid, while > 0 was regarded as amplified. RSEM-normalized RNA-seq values were log2 transformed before analysis. Among these 1014 primary tumors, PAM50 subtypes were determined in 500 samples, in which normal-like subtype containing only 8 samples was excluded from further analysis.

### Two-step geomapping method

Pearson correlation and optimal univariate *k*-means clustering methods were applied in the geomapping approach to identify amplification-associated transcription coupling loci. In the first step, we used 1014 tumor samples from TCGA cohort and surveyed all genes in chromosome 17 that displayed high correlation between their expression and copy number levels. Only genes showing a positive correlation coefficient ≥ 0.6 were selected in our analysis. In the next step, the *k*-means clustering method was applied to target the aggregation of these analyzed genes using *Ckmeans.1d.dp* package (version 4.2.2) in R [17]. We initially identified 11 clustered regions, but only seven gene-rich regions were selected for the downstream analysis based on the following criteria: 1) containing more than or equal to 15 genes; 2) less than 20 Mb in the total length; and 3) with density estimates higher than 1.0 × 10^− 08^.

### Identification of chromosomal rearrangements and dense ERα binding sites

Inter−/intra-chromosomal rearrangements were detected by BreakDancer [18] with parameter “-t” using whole-genome DNA sequencing data of TCGA breast cancer cohort from Cancer Genomics Hub. The output events with confidence scores higher than 80 were used in the downstream analysis for quality control purpose. The filtered events of inter−/intra-chromosomal rearrangements were visualized using Circos with 1-Mb as unit [19]. A total of 170 dense ERα binding sites were defined by univariate *k*-means clustering methods using *Ckmeans.1d.dp* package in R (Additional file 2: Table S1). ERα ChIP-seq data of three cell lines were downloaded from Cistrome Data Browser [20].

### Cell cultures, chemicals, and growth and clonogenic assays

Human breast cancer cell lines MCF-7 (HTB-22), BT20 (HTB-19), BT474 (HTB-20), MDA-MB-157 (HTB-24), MDA-MB-231 (HTB-26), and MDA-MB-361 (HTB-27), and benign breast cell lines MCF10A (CRL-10317) and MCF12A (CRL-10782) were obtained from ATCC and cultivated in DMEM supplemented with 10% FBS at 37 °C and 5% CO_2_. Cell authentication was conducted at ATCC by using short tandem repeat DNA profiling. Human mammary epithelial cells (HMEC, Cat# A10565) were obtained from ThermoFisher. Perphenazine (Sigma-Aldrich, P6402), trifluoperazine (Sigma-Aldrich, T8516), thioridazine (Sigma-Aldrich, T9025), and bleomycin (Sigma-Aldrich, 203408) were purchased from Sigma-Aldrich. The drugs were dissolved in ethanol with a final concentration of 0.025% (v/v). Concentration-matched controls were used in the drug experiments.

Cell growth was also assessed by measuring cell confluence using IncuCyte ZOOM live-cell analysis system (Essen BioScience). Cells were seeded overnight in 96-well plates at a density of 1,000–5,000 cells per well and growth curves were generated by imaging every 12 h with quadruplicate replicates. Cell viability was quantified using CellTiter-Glo reagent (Promega) according to the manufacturer’s instructions. Cells were plated at a density of 1,000 cells per well in 96-well plates and allowed to settle overnight. Cells were treated for 3 days before cell viability was measured. Cell lysis was induced by mixing for 30 min on an orbital shaker and plates were then incubated at room temperature for 10 min to stabilize luminescent signal. Luminescence readout was performed on Luminoskan Ascent microplate luminometer (Thermo Fisher Scientific). The amount of light measured was expressed in relative light units (RLU).

For clonogenic assays, cells were seeded at a density of 5,000 cells per well in 6-well plates and allowed to adhere overnight in regular growth media. Cells were then cultured in the absence or presence of drug as indicated in complete media for 10–14 days. Growth media with or without drug was replaced every 3 days. Remaining viable cells were fixed with 4% paraformaldehyde and stained with 0.5% crystal violet in 20% methanol (Sigma-Aldrich). Relative growth was quantified by densitometry after extracting crystal violet from the stained cells using 10% of acetic acid.

### siRNA knockdown

MCF-7 cells were transfected with siRNA duplexes to target *TLK2* (Ambion, s21679) using Lipofectamine RNAiMAX transfection reagent (Invitrogen) as per the manufacturer’s recommendations, and incubated for 48 h, followed by protein extraction for Western blot analysis. Silencer Select negative control siRNA (Ambion, AM4611) was used as a non-targeting control.

### Nanopore sequencing

Translocations between chromosomes 17q23 and 20q13 in MCF-7 cells were detected using Nanopore sequencing. Genomic DNA was subjected to whole-genome amplification (WGA) using REPLI-g Midi kit (Qiagen) and purified as per manufacturer’s recommendations. Barcoded libraries were then constructed with WGA DNA, quantified using Qubit dsDNA HS assay reagent (Invitrogen), normalized, and pooled to a final amount of 1 μg. After end-repair and dA-tailing using NEBNext Ultra II end-repair/dA-tailing module (New England Biolabs), libraries were subjected to ligation of hairpin and leader adapters using SEQ-NSK-007 sequencing kit (Oxford Nanopore Technologies), followed by loading onto Nanopore flow cell FLO-MIN104 and sequencing on MinION Mk1B device (Oxford Nanopore Technologies) for up to 36 h. Alignments were performed against NCBI hg38/GRCh38 using LAST aligner [21] with the parameters “lastal -Q1 -r5 -q5 -a30 -b5 -e100”. Visual outputs were obtained from searches using NCBI BLAST of Nanopore 2D reads against hg38/GRCh38 using default parameters. Primer sequences used in Nanopore sequencing are listed in Additional file 2: Table S2.

### CRISPR/Cas9 editing and RT-qPCR

To delete the ERα-bound enhancers at 20q13 from the genome, MCF-7 cells were transfected with plasmids containing guide RNAs (GeneCopoeia) targeting the left and right sides of the 1-kb region encompassing the eight ERα binding sites [22]. Colonies were derived from single cells and validated for the depletion of the enhancer cluster region as previously described [22]. To minimize the influence of endogenous estrogen, cells were cultured in phenol red-free DMEM containing 0.2% charcoal-stripped FBS for 24 h prior to assays. Cells were then treated with 70 nM E2 or dimethylsulfoxide (DMSO) vehicle for 24 h. Total RNA was extracted using Quick-RNA MiniPrep kit (ZYMO Research) with in-column DNA digestion following the manufacturer’s standard protocol. Reverse transcription of 2 μg RNA was performed using SuperScript VILO Master Mix (Invitrogen) and random hexamers (Promega). Quantitative RT-PCR was carried out with PowerUp SYBR Green Master Mix on 7900HT Fast Real-Time PCR System (Applied Biosystems). After 40 cycles of amplification, melt curves were examined to ensure primer specificity, and relative expression of each gene was calculated after normalizing to *GAPDH* expression by applying the *ΔΔ*Ct comparative quantification method. Primer sequences used in CRISPR/Cas9 experiments are provided in Additional file 2: Table S3.

### Copy-number analysis

Genomic DNA was extracted from breast cancer and non-neoplastic epithelial cells using Gentra Puregene kit (Qiagen), followed by additional ethanol precipitation, a rinse with 70% ethanol, air drying, and resuspension in hydration buffer. RNase A and proteinase K digestion was included in the isolation procedure according to the manufacturer’s instructions. The quality and concentration of isolated genomic DNA was evaluated using NanoDrop 2000 (Thermo Fisher Scientific) and each DNA sample was routinely assessed by agarose gel electrophoresis with GelRed staining to ensure the absence of contaminating RNA and degradation of genomic DNA. Amplification was performed on StepOne Plus instrument (Applied Biosystems) using PowerUp SYBR Green Master Mix (Applied Biosystems). Relative copy number was determined in triplicate by comparing Ct values for the primer set at the 5′-end of the *TLK2* locus to those for the reference *RPPH1* gene [23]. Primer sequences used in copy-number analysis are listed in Additional file 2: Table S4.

### Western blotting

Cells were washed with ice-cold PBS and lysed on ice for 30 min with lysis buffer containing 20 mM Tris-HCl, 1% NP-40, 150 mM NaCl, 10% glycerol, and protease inhibitor cocktail (Thermo Fisher Scientific). After clearing by centrifugation, protein concentration was determined by Bradford protein assay kit (Bio-Rad) and a calibration standard curve created from bovine serum albumin. Total proteins were separated by SDS-PAGE before being electrophoretically transferred onto a PVDF membrane (Bio-Rad). Membranes were blocked in 2% goat serum/0.5% skimmed milk in PBST (0.05% Tween 20) and then probed with primary antibodies overnight at 4 °C. After incubation with secondary antibodies, membranes were incubated with Clarity Western ECL substrate (Bio-Rad). Primary antibodies used in Western blot analysis were as follows: mouse anti-TLK2 (1:1000, sc-393,506, Santa Cruz Biotechnology), rabbit anti-γH2AX (1:2000, 9718, Cell Signaling Technology), rabbit anti-RAD51 (1:1000, sc-8349, Santa Cruz Biotechnology), rabbit anti-phospho-p53 (1:1000, 9284, Cell Signaling Technology), mouse anti p53 (1:500, MA5–12557, Thermo Fisher Scientific), and mouse anti-α-tubulin (1:2000, sc-8035, Santa Cruz Biotechnology).

### Ex vivo culture of circulating tumor cells

CD45-negative circulating tumor cells (CTCs) were obtained from peripheral blood samples of breast cancer patients as described previously [24] with modifications (Additional file 2: Table S5). Briefly, peripheral blood (~ 8 mL) was collected in EDTA tubes and red blood cells were removed by adding RBC lysis buffer (v/v: 1/6, ScreenCell), followed by separation with Ficoll-Paque density gradient (GE HealthCare). After exclusion of CD45-positive cells by magnetic beads (StemCell Technologies), cells were plated on ultra-low attachment 24-well plates (Corning Costar) and maintained for 1–2 weeks in PRIME XV Tumorsphere medium (Irvine Scientific) supplemented with 2.0 U/ml of heparin (Sigma-Aldrich) and 0.5 μg/mL of hydrocortisone (Sigma-Aldrich). The study was reviewed and approved by the Institutional Review Board of the University of Texas Health Science Center at San Antonio and patients provided written informed consent.

### Immunofluorescence analysis

Cells were fixed with 4% paraformaldehyde for 10 min and permeabilized with 0.5% Triton X-100 for 5 min at room temperature. Nonspecific binding was blocked with 5% goat serum in PBST (0.1% Triton X-100 in PBS) for 30 min at room temperature. Immunostaining was performed at 4 °C overnight with primary antibodies, followed by fluorescence-conjugated secondary antibodies (Invitrogen). Images were captured with Zeiss LSM710 confocal microscope at CSA Optical Imaging Facility. Primary antibodies used in immunofluorescence staining were as follows: mouse anti-γH2AX (1:400, 05–636, Millipore), mouse anti-pan-CK (1:3000, 4545, Cell Signaling Technology), rabbit anti-EpCAM (1:200, ab71916, Abcam), mouse anti-CD45 (1:200, ab33533, Abcam), rat anti-CD44 (1:400, ab40983, Abcam), rabbit anti-ALDH1 (1:200, bs-10162R, Bioss), rabbit anti-NANOG (1:800, ab21624, Abcam), mouse anti-OCT4 (1:500, ab184665, Abcam).

### Statistical analysis

Heatmaps were plotted with *heatmap.2* function in *gplots* package (version 3.0.1.1) and dot plots were made with *ehplot* function in *plotrix* package (version 3.7–5) in R. Multiple comparison between the expression of ERα-positive, −negative and normal controls and between PAM50 subtype samples was employed with *PostHocTest* function in *DescTools* package (version 0.99.28). Gene-gene correlation matrices were calculated based on expression level/copy number data of the genes in the 5 target regions of chromosome 17. Values of the correlation coefficients of the genes were presented symmetrically in the heat maps using *corrplot* package (version 0.84) in R. Kaplan Meier overall survival curves in ERα-positive samples were made with *ggsurvplot* in *survminer* package (version 0.4.3) in R. Between-group comparison of mean values was performed with Mann-Whitney U test or one-way ANOVA with Dunnett post-hoc test using GraphPad Prism 7. All results are given as mean ± standard deviation (SD) unless indicated otherwise. *P*-values of 0.05 or less were considered statistically significant.

## Results

### Genomic survey identifies 177 putative ATC loci on chromosome 17 regions

**Fig. 1:**
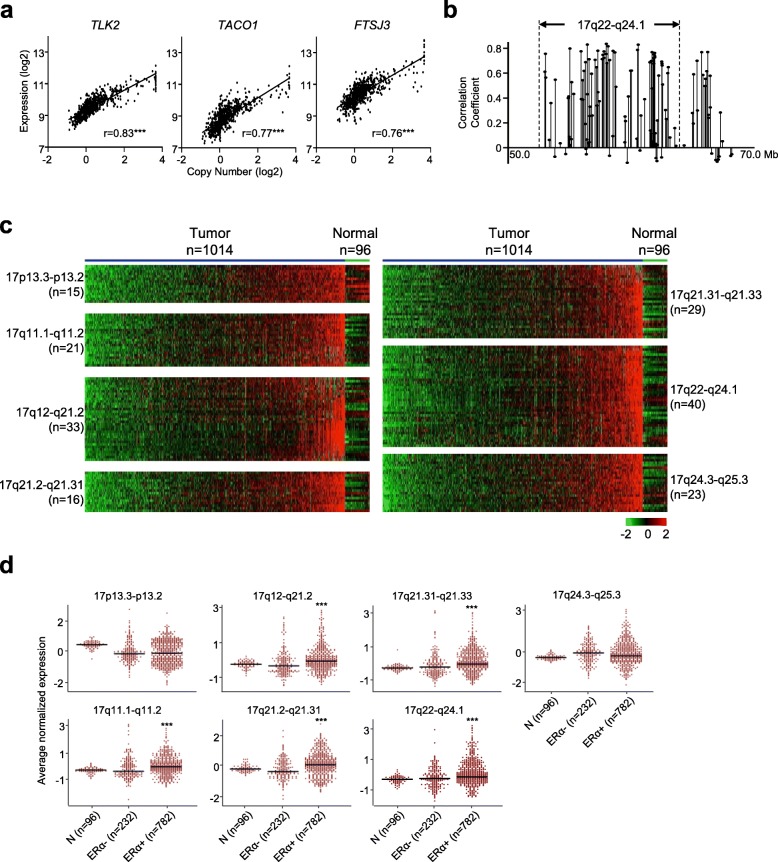
Genomic survey with two-step geomapping approach identifies amplification-associated transcription coupling (ATC) loci clustered in seven genomic regions. **a**. Example genes with high correlation coefficients between copy-number and expression levels. **b**. Example region aggregated with genes of expression and copy-number correlation coefficients ≥0.6 (referred as ATC loci) identified by the optimal univariate *k*-means clustering method. **c**. Expression profiles of ATC loci in the seven identified genomic regions by tumor (*n* = 1014) and normal controls (*n* = 96). **d**. Average normalized expression of ATC loci in the seven genomic regions by the status of estrogen receptor α (ERα). Pearson correlation coefficients (r) and *P*-values are shown (*P < 0.05, **P < 0.01, ***P < 0.001)

Considering that the expression of genes geographically located within close neighborhoods can be co-regulated by the same set of transcription factor machinery, we conducted a comprehensive survey of chromosome 17 using the Cancer Genome Atlas (TCGA) breast cancer cohort data to map multiple loci undergoing amplification-associated transcription coupling (ATC). We adopted a two-step geomapping approach by integrating the methods of Pearson correlation and optimal univariate *k*-means clustering. In the first step, we screened loci contiguously localized on genomic regions that displayed high correlation between expression and copy-number in 1014 TCGA tumors. A correlation value of ≥0.6 for a given locus was considered to ensure high association (Fig. 1a). The optimal univariate *k*-means clustering method then identified expression aggregations based on genomic locations of amplified loci (Fig. 1b). This approach found a total of 177 putative ATC loci, clustered within seven genomic regions (average 6.9-Mb in length) on chromosome 17 (Fig. 1c).

The presence of ATC implicated that loci located within close neighborhoods could be co-regulated by the same set of transcription machinery. Based on average expression levels of genes in these genomic regions, we found that ATC loci were frequently linked to hormone-receptor status of breast tumors. Specifically, preferential upregulation of ATC loci localized on five adjacent regions (i.e., 17q11.1, 17q12, 17q21.2, 17q21.31, and 17q22) occurred more frequently in ERα-positive tumors relative to ERα-negative tumors and normal controls (*P* < 0.001, Fig. 1d). However, ATC loci of the other two regions (i.e., 17p13.3 and 17q24.3) displayed no statistical differences of the expression levels between ERα-positive and -negative tumors (Fig. 1d). Supporting our previous findings [6, 22], this result suggests that a subset of ATC loci is coordinately activated or repressed in part by the ERα regulatory elements possibly through nearby intra-chromatin looping or distant inter-chromatin interactions.

### ATC loci localized on 17q11.1-q24.1 are functionally linked to cell-cycle control

**Fig. 2:**
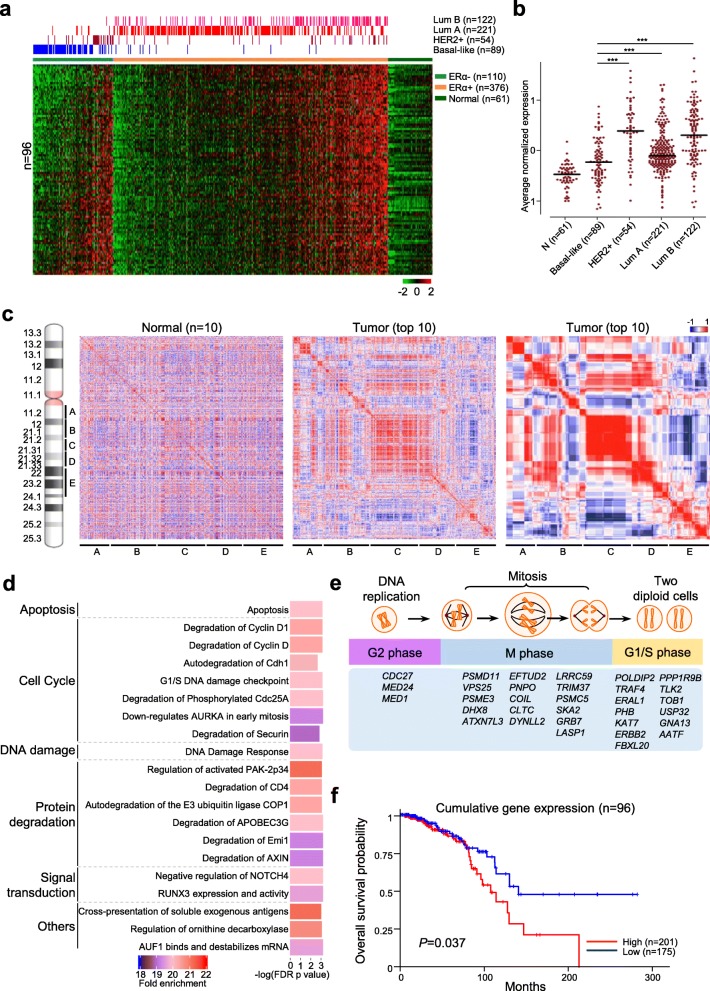
Amplification-associated transcription coupling (ATC) loci on 17q are functionally linked to cell-cycle control. **a**. The expression profile of breast tumors with PAM50 subtypes and normal controls of the 96 ATC loci from the five regions on 17q (17q11.1-q11.2; 17q12-q21.2; 17q21.2-q21.31; 17q21.31-q21.33; 17q22-q24.1). Samples of PAM50 subtype status are shown in different colors on the top of the heat map. Details of gene names were provided in Additional file 1: Figure S1. **b**. Average normalized expression of the 96 ATC loci in different PAM50 subtypes and normal controls. *P*-values are shown (***P < 0.001). **c**. Locations of the five regions indicated on the ideogram of chromosome 17. Correlation profiles of all genes in the five 17q regions calculated based on their expression levels (*left* and *middle*) and copy numbers (*right*). The genes were listed symmetrically in each profile (from left to right and from top to bottom) according to the order of their genomic locations. Normal controls were randomly selected from the 61 adjacent normal samples in Fig. 2A, and tumor groups were 10 samples with the highest overall expression of the 96 ATC loci. The locations of the five genomic regions were indicated beneath the correlation profiles. **d**. Pathway enrichment analysis of the 96 ATC loci using the GO enrichment analysis tools. **e**. Thirty-two genes among the 96 ATC loci are related to cell-cycle functions based on literature review. **f**. Kaplan-Meier overall survival curves of ERα-positive samples grouped by high/low cumulative normalized expressions of the 96 ATC loci. *P*-values were calculated using log-rank tests

Of 139 ATC loci identified in the five adjacent regions on 17q, we found that the expression of 96 putative loci was consistently upregulated in breast tumors than normal controls (Fig. 2a). Moreover, their expression levels appeared higher in luminal breast tumors, especially luminal B subtype, than in basal-like tumors (*P* < 0.001, Fig. 2b, Additional file 1: Figure S1). This trend was also observed in breast tumors with co-amplification of *human epidermal growth factor receptor 2* (*HER2*) and other ATC loci mapped on 17q12-q21.2 [25]. Although these 96 ATC loci only accounted for a small number (17%) of all 572 protein-coding genes mapped on the five regions A, B, C, D, and E (17q11.1-q11.2, 17q12-q21.2, 17q21.2-q21.31, 17q21.31-q21.33, and 17q22-q24.1, respectively), their expression levels actually reflected high degrees of gene-gene correlation in breast tumors (e.g., top 10 selected from the TCGA cohort, Fig. 2c, *middle*). Copy-number changes were also highly correlated in the adjacent regions, further confirming concordant regulation of the 96 ATC loci in tumors (Fig. 2c, *right*).

Pathway enrichment analysis indicated that biological functions of the 96 ATC loci are highly related to cell cycle, DNA damage, apoptosis, and protein degradation (Fig. 2d). Specifically, there were 32 loci encoding cell cycle-related proteins responsible for the assembly of anaphase promoting complex [26], mitotic entry and spindle assembly [27], DNA damage checkpoints at G1/S phase [28], and maintenance of G1 arrest [29] (Fig. 2e). A Kaplan-Meier plot displayed that cumulatively high expression levels of the 96 ATC loci were linked to poor overall survival of a subset of 376 ERα-positive breast cancer patients in the TCGA cohort (*P* = 0.037, Fig. 2f).

### ERα-related chromosomal rearrangements are detected in five 17q regions of ATC loci

**Fig. 3:**
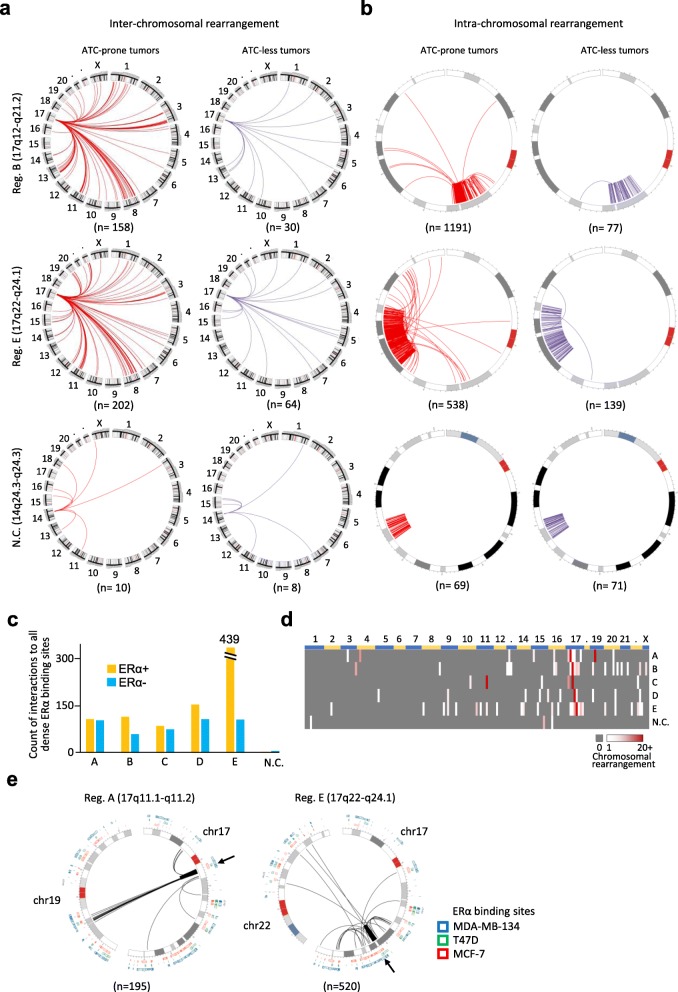
ERα-related chromosomal rearrangements of the five 17q regions. **a**, **b**. Comparison of frequencies of chromosomal rearrangements between ATC-prone tumors (*n* = 15) and ATC-less tumors (*n* = 15). Circos plots of inter- (**a**) and intra- (**b**) chromosomal rearrangements related to two 17q regions and one negative control region on 14q. The plots of the rest 17q regions were displayed in Additional file 1: Figure S2. **c**. Frequencies of inter−/intra-chromosomal rearrangements in the five 17q regions by the hormone receptor status. **d**. Frequencies of inter−/intra-chromosomal rearrangements of each 17q regions against the 170 dense ERα binding sites of the whole genome. The dense ERα binding sites were ordered based on their genomic locations in each chromosome on the X-axis of the heat map (also see Additional file 2: Table S1). **e**. Examples of inter−/intra-chromosomal rearrangements against specific dense ERα binding site from two 17q regions (Region A and Region E pointed by black arrows). Locations of ERα binding sites from MCF-7, T47D, and MDA-MB-134 were displayed outside around the Circos plots

Knowing that ATC loci could be hotspots for non-random insertions of distant *trans*-regulatory elements [6], we used TCGA DNA sequencing data of 96 breast cancer samples with sufficient sequencing depth to analyze the occurrence of inter−/intra- chromosomal rearrangements, i.e., an indicator of chromothripsis, related to the five 17q regions. The association between chromosomal rearrangements and the concordant levels of ATC loci was first examined (Fig. 3a, b, and Additional file 1: Figure S2). Representative samples of 15 ATC-prone tumors (with the highest concordant expression levels) and 15 ATC-less tumors (with the lowest concordant expression levels) were selected based on the total expression levels of 96 loci in the heat map of Fig. 2a. Using the five 17q regions as “baits” to identify their interacting partners, we found more rearrangements in the ATC-prone tumors than the ATC-less tumors in both inter- and intra-chromosomal rearrangements. As a negative control, a region on 14q24.3-q24.3 where no ATC loci existed showed very few rearrangements. The finding confirmed that high frequencies of genomic rearrangements were related to concordant over-expression of the ATC loci in the five regions analyzed. Among these regions, regions B and E displayed high numbers (*n* = 158 and 202, respectively) of inter-chromosomal rearrangements with sub-regions of chromosomes 1, 3, 8, 11, 13 and 20 in these ATC-prone tumors. The numbers of intra-chromosomal rearrangements were even larger (*n* = 1191 and 538, respectively), compared to those inter-chromosomal events. These results suggest that regions B and E are susceptible to chromothripsis in ATC-prone tumors.

Considering the hormone receptor status of the samples, our data revealed that the frequency of both inter- and intra-chromosomal rearrangements was preferentially higher in ERα-positive tumors than in ERα-negative tumors (see also a distinct event of region E in Fig. 3c). The high frequency of chromothripsis in these regions corroborated the increased expression levels of ERα mediated genes in a group of luminal breast tumors (Fig. 2b). To further survey 17q-related chromothriptic events, we defined a total of 170 dense ERα binding sites across the whole genome, with an average length of 2-Mb based on common ERα binding sites detected from MCF-7, T47D, and MDA-MB-134 breast cancer cell lines [30–32] (Fig. 3d and Additional file 2: Table S1). On average, around 270 ERα-related genomic rearrangements (both inter- and intra-events) were observed in the five 17q regions, with the highest of 544 events on 17q22-q24.1 (Region E in Fig. 3d). As expected, nine of these dense ERα binding sites (or ERα hubs) on chromosome 17 drew extensive intra-chromosomal rearrangements with the five aforementioned regions, accounting for ~ 90% of ERα-related chromothripsis in the whole genome. Noted that non-random inter-chromosomal rearrangements were observed between our target regions on 17q and particular ERα hubs on other chromosomes. For example, region A (17q11.1-q11.2) showed frequent rearrangements with one dense ERα binding region on chromosome 19p13.12, and the same situation was detected between region C (17q21.2-q21.31) and chromosome 11q14.1. Detailed inter−/intra-chromosomal rearrangements of two regions A and E were further displayed in Circos plots as examples (Fig. 3e). It is clear that the two regions were also located within dense ERα binding regions. Region A had frequent rearrangements with a dense ERα hub on chromosome 19p13.12 whereas Region E showed multiple rearrangements with dense ERα binding sites on chromosome 22. Due to the limitation of visualization unit (1-Mb), some geographically similar rearrangements may be displayed in the same line in the plots, but the exact numbers of rearrangements were shown under the plots.

### The transcription of 17q23 ATC loci is concordantly regulated through long-range interactions with an ERα hub on 20q13

**Fig. 4:**
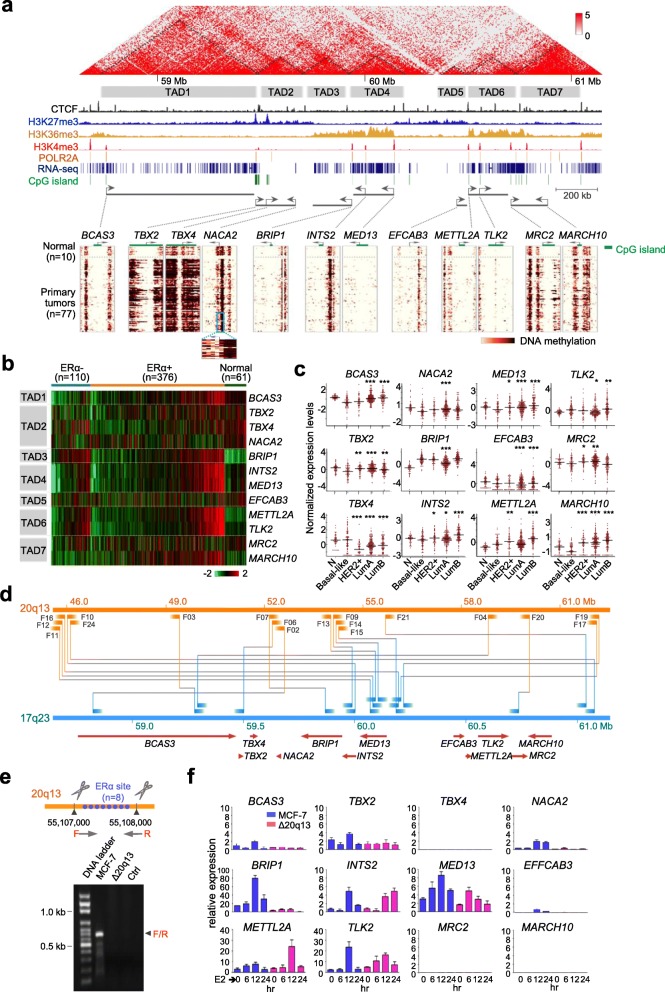
The transcription of 17q23 amplification-associated transcription coupling (ATC) loci is concordantly regulated through long-range interactions with an ERα hub on 20q13. **a**. 17q23 region with Hi-C interaction frequencies overlaid on ChIP-seq peaks and RNA-seq (*top*) from human mammary epithelial cells (HMECs), and DNA methylation landscapes (*bottom*) of primary breast tumors and normal controls. The dotted triangles indicate identified topologically associating domains (TADs). A total of seven TADs (four active/open and three repressive/closed) at the 17q23 region flanked by enrichment of boundary-associated CTCF peaks. **b**. Expression profile of the 12 genes in this region of tumors and normal controls. **c**. Normalized expression of the 12 genes on 17q23 by PAM50 subtypes. **d**. Inter-chromosomal rearrangement between 17q23 and 20q13. Breakpoint junctions used for Nanopore sequencing (see also Additional file 1: Figure S3). **e**. Abrogation of estrogen-mediated transcriptional activation of 17q23 genes by CRISPR/Cas9 targeted deletion of an ERα hub at 20q13. Blue dots indicate previously identified ERα sites located within a 1-kb region on 20q13. Arrowheads denote the targeting sites by the single guide RNAs (sgRNAs) and PCR primers used for validation of the deletion are shown by horizontal arrows. **f**. Quantitative RT-PCR carried out upon estrogen (E2) stimulation over a time course. Data shown are mean ± SD of three independent experiments

Next we determined how the expression of ATC loci is regulated in topologically associating domains (TADs) that can be partitioned into active and inactive compartments in large contiguous chromosome regions [33]. To further elucidate this co-regulatory event, we specifically characterized TADs and related histone marks in a sub-region of one of the aforementioned five regions, i.e., 17q22-q24.1. This region at 17q23 was grouped into seven TADs that are flanked by boundary-associated CTCF binding sites in human mammary epithelial cells (HMECs, Fig. 4a, *top*) [33, 34]. Four ATC loci - *INTS2*, *MED13*, *METTL2A* and *TLK2* were localized on active TAD4 and TAD6, marked by H3K36me3 for actively transcribed gene bodies and by H3K4me3 for active promoters (Fig. 4a, *middle*) [35], which was also reflected on RNA-seq peaks. Although not specified as ATC loci based on a correlation value ≥0.6, *BCAS3* and *BRIP1* were also found to be localized within active TAD1 and TAD3, respectively. There were six non-ATC loci - *TBX2*, *TBX4*, *NACA2*, *EFCAB3*, *MRC2* and *MARCH10* located separately on three repressive domains, i.e., TAD2, TAD5, and TAD7, which were enriched with the repressive chromatin mark H3K27me3.

We then assessed DNA methylation patterns across the 17q23 region using our previous breast cancer cohort based on methyl-binding domain capture sequencing [36]. Located within the inactive TADs, five of the six aforementioned non-ATC loci (except *EFCAB3*) were highly methylated at shore regions of CpG island promoters (i.e., *TBX2*, *TBX4*, *MRC2*, and *MARCH10*) and non-CpG island promoter (i.e., *NACA2*) in 77 breast tumors compared to 10 normal controls (Fig. 4a, *bottom*; ref. *#* [37]). This regional increase in DNA methylation is likely attributed to enhanced activities of DNA methyltransferases during tumorigenesis [38]. However, the shore methylation seemed not to have an adverse effect on repressing basal transcription in tumors. As transcription start sites of the four loci remained unmethylated for transcription factor binding, low expression levels observed in normal controls could sporadically be detected in TCGA breast tumors (Fig. 4b). On the contrary, the four ATC loci residing in the active TAD4 and TAD6 exhibited very low methylation levels in their neighboring regions (Fig. 4a, *bottom*). As expected, concordantly high expression levels of these loci were observed in more than 20% of 376 ERα-positive tumors, compared to those of normal controls. Although less concordant, elevated expression of the loci was occasionally observed in ERα-negative tumors (Fig. 4b). Our observation suggests that the expression of individual loci localized within amplified genome segments can be co-modulated in a permissive chromatin topological landscape. When comparing the expression levels of these 12 genes across breast cancer subtypes, the results showed most of the genes displayed higher expressions in luminal subtypes compared with a basal-like subtype, which suggests the transcription in this region is highly affected by estrogen stimulation (Fig. 4c).

Knowing that the 17q23 region is within one of the five regions that showed frequent inter−/intra-chromosomal rearrangements, we used nanopore sequencing for long-range genome mapping and independently confirmed frequent rearrangements of 18 junctions between ATC loci on 17q23 and an ERα hub on 20q13 in MCF-7 luminal breast cancer cells (Fig. 4d and Additional file 1: Figure S3). To further determine a functional relationship between ATC loci and ERα hubs, the CRISPR/Cas9 editing was used to delete a 1-kb region that harbors eight 20q13 ERα binding sites in MCF-7 cells (Fig. 4e). As a result, synchronized control for the expression of nine out of 12 genes located on 17q23 was disrupted after edited cells were stimulated with estrogen (E2) for a period of 24 h (Fig. 4f). The deregulation was more profound in the aforementioned four ATC loci - *INTS2*, *MED13*, *METTL2A* and *TLK2*, as well as in *BCAS3* and *BRIP1*, which might be concordantly regulated by ERα to a lesser extent. Taken together, our findings further establish that the 20q13 region can serve as an ERα hub to remotely regulate the expression of ATC loci on 17q.

### The ATC locus *TLK2* is a potential actionable target for luminal-HER2 cancers

Herceptin and its derivatives have been used to treat breast tumors with amplified *HER2* (or *ERBB2*) on 17q, but resistance to these therapies frequently develops in patients [39, 40]. While the underlying mechanisms are complex, other ATC loci located in the region might contribute to this resistance especially for the luminal-HER2 subtype that is both ERα-positive and HER2-positive [41]. To search for an additional druggable target on 17q, we found an ATC locus, *Tousled Like Kinase 2* (*TLK2*) as a potential candidate for aggressive luminal tumors [42, 43]. *TLK2* silencing by shRNA suppressed tumor proliferation and invasion in vitro and in xenograft studies [43]. However, small molecule inhibitors to counter TLK2 activities were not applicable in vivo due to off-target effects [43].

**Fig. 5:**
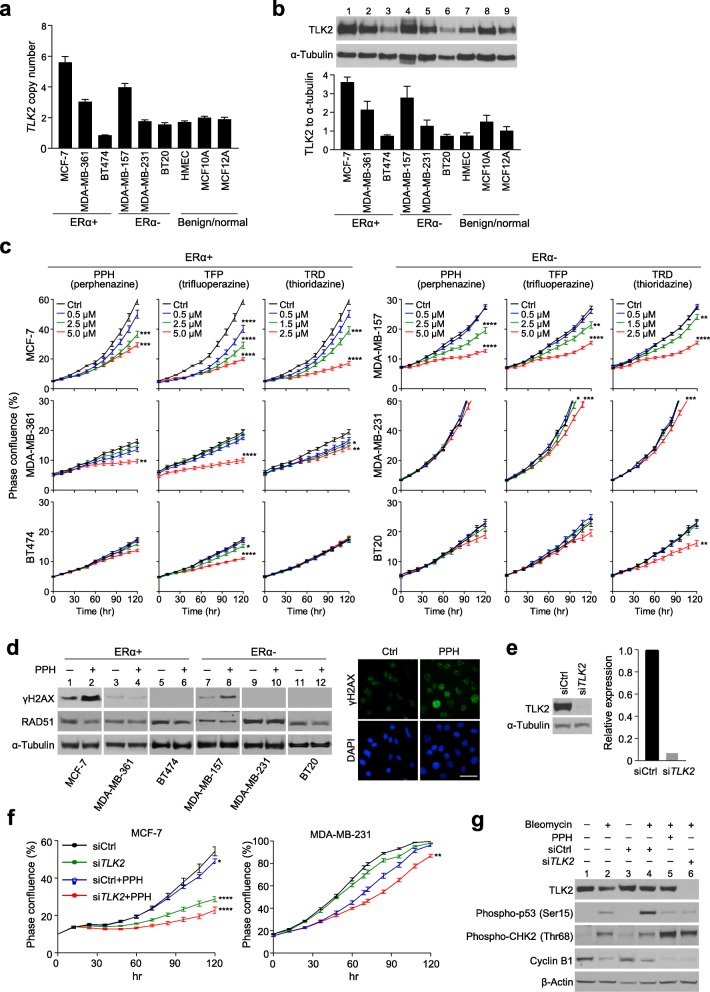
The amplification-associated transcription coupling (ATC) locus TLK2 is an actionable target for 17q23-amplified breast cancers. **a**. DNA copy-numbers of *TLK2* across normal, benign and breast cancer cell lines. Quantitative PCR assays were used to determine copy number alterations. Data represent mean ± standard error of the mean (SEM) of three independent experiments. **b**. Western blot detecting TLK2 protein expressed in normal, benign and breast cancer cell lines. Loading amount was normalized with α-tubulin. Data represent mean ± standard error of the mean (SEM) of three independent experiments. **c**. Antiproliferative effects of antipsychotics on breast cancer cell lines. Cells were treated with or without phenothiazine derivatives (PPH, TFP and TRD) at various concentrations. Phase confluence percentage of the cells was collected every 12 h totally for a period of 5 days using IncuCyte ZOOM live-cell imaging system. Quadruplicate replicates were used in each experiment. Quantitative analysis of cell growth 120 h after drug treatment by one-way ANOVA (vs Ctrl). **d**. DNA damage response in cells treated with or without PPH (5.0 μM) was analyzed with Western blot and immunostaining. Double strand breaks were induced by bleomycin. γH2AX was accumulated more in MCF-7 and MDA-MB-157 cells treated with PPH. No significant difference of RAD51 expression was noted between the cells treated with or without PPH. Representative immunofluorescence images of γH2AX foci (green) were shown in MCF-7 cells treated with or without PPH (*right*). Scale bar, 30 μm. **e**. Western blot analysis of *TLK2* siRNA knockdown efficiency in MCF-7 cells. TLK2 protein level was markedly decreased in the cells treated with *TLK2* siRNA. **f**. Inhibitory effect of *TLK2* knockdown and PPH treatment (5.0 μM) on cell proliferation of MCF-7 and MDA-MB-231 cells. Phase confluence percentage of the cells was collected every 12 h totally for a period of 5 days using IncuCyte ZOOM live-cell imaging system. Quantitative analysis of cell growth 120 h after drug treatment by one-way ANOVA (vs siCtrl). **g**. Western blot analysis of the indicated signaling molecules in MCF-7 cells treated with bleomycin, *TLK2* siRNA knockdown and/or PPH

An alternative approach was phenothiazine antipsychotics (PTZs) previously shown to be potential inhibitors of TLK family proteins by disrupting their ability to phosphorylate cell-cycle checkpoint regulators [44]. Therefore, we assessed the growth-inhibitory effect of three PTZs in a panel of nine breast cancer and immortalized cell lines exhibiting respective copy-number and protein levels of *TLK2* (Fig. 5a and b). Antiproliferation was prominent at 5.0 μM of perphenazine (PPH) and trifluoperazine (TFP) or at 2.5 μM of thioridazine (TRD) for high TLK-expressing cell lines MCF-7, MDA-MB-361, and MDA-MB-157 (*P* < 0.01, *P* < 0.001 and *P* < 0.0001, Fig. 5c). Low TLK2-expressing cell lines BT474, MDA-MB-231 and BT20 were relatively insensitive to PTZs, which also showed no inhibitory effect on three immortalized cell lines tested (data not shown).

Since TLKs were implicated in the process of DNA damage response (DDR) [45], we examined the effect of PTZs on bleomycin-induced DDR signaling. Because TFP and TRD were discontinued for clinical treatments due to side effects [46, 47], we focused on PPH for the study. PPH treatment resulted in increased phosphorylated H2AX (γH2AX) in high TLK2-expressing MCF-7 and MDA-MB-157 cells (lanes 2 and 8, Fig. 5d, *left*), but γH2AX was not readily detectable in the remaining cell lines. Immunofluorescence staining further confirmed a marked increase in DNA double-strand breaks (DSBs) upon PPH exposure in high TLK-2 expressing MCF-7 cells (Fig. 5d, *right*). To further determine whether this antitumor activity is an on-target consequence of TLK2 inhibition, we used siRNA-mediated knockdown (KD) of *TLK2* (Fig. 5e), followed by the treatment with or without PPH. *TLK2* KD alone was sufficient to inhibit proliferation of MCF-7 cells, and similar growth suppression was also observed in cells with KD plus PPH treatment (*P* < 0.0001, Fig. 5f, *left*). Compared with MCF-7 cells, MDA-MB-231 cells with low TLK2 were less sensitive to the single or combined treatment (Fig. 5f, *right*). Further analysis indicated that phosphorylation of both cell-cycle checkpoint protein p53 and DDR protein CHK2 was induced in response to bleomycin treatment (Fig. 5g, lanes 2 and 4); however, a similar decrease in phospho-p53 was found in both TLK2 KD and PPH-treated cells, whereas increased phospho-CHK2 was present in these two conditions (Fig. 5g, lanes 5 and 6). These observations suggest that selective inhibition of TLK2 contributed to decreased cell proliferation (see also reduced Cyclin B1 level in Fig. 5 g) at least in part through targeting of TLK2-mediated DDR pathway.

**Fig. 6:**
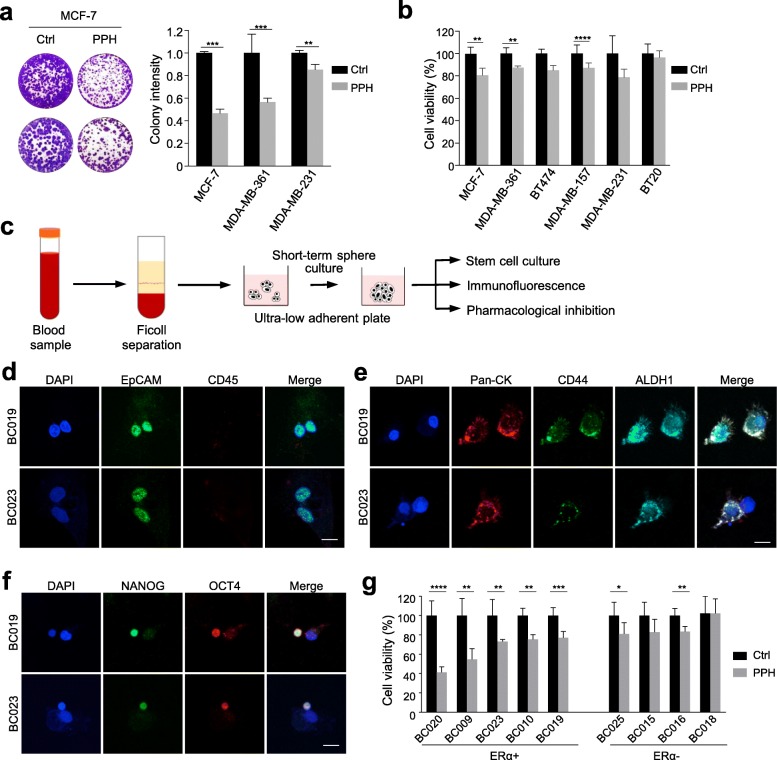
Ex vivo pharmacological treatment reduces survival of breast cancer cells. **a**. Clonogenic assays show long-term effect of PPH treatment on MCF-7, MDA-MB-361, and MDA-MB-231 cells. Colony intensity was shown as mean ± SD of three replicates. **b**. Cell viability assay on breast cancer cell lines after treatment with PPH for 72 h. Data are represented as mean ± SEM of five replicates. **c**. Workflow of isolation, enrichment and downstream analysis of circulating tumor cells (CTCs) from breast cancer patients’ blood samples. **d-f**. Immunostaining of enriched CTCs derived from patient blood samples. Scale bars, 10 μm. **g**. Cell viability assay of patient CTCs with PPH treatment. Enriched CTCs were treated with 5 μM of PPH or vehicle control for 72 h. Data are represented as mean ± SEM of five replicates. (*P < 0.05, **P < 0.01, ***P < 0.001, and ****P < 0.0001, Student’s t-test)

We also tested the ability of cancer cells to form colonies in the presence or absence of PPH. As expected, PPH exposure led to a decrease in colony formation ability, and the effect was more apparent in high TLK2-expressing cells (MCF-7 and MDA-MB-361) than in low TLK2-expressing MDA-MB-231 cells (Fig. 6a). The finding was consistent with the cell survival assay showing a significant decrease in cell viability in all three high TLK2-expressing cancer cells (Fig. 6b). To extend the potential use of TLK2 inhibitor in clinical samples, we examined the efficacy of PPH on ex vivo cultures of circulating tumor cells (CTCs) isolated from blood samples of nine breast cancer patients (Fig. 6c). Immunofluorescence staining of these cells confirmed their epithelial origins (e.g., EpCAM+, pan-CK+ and CD45-) and stem-cell properties (e.g., ALDH1+, NANOG+, and/or OCT4+) (Fig. 6d-f). Cell viability assays of 5 μM PPH treatment demonstrated that CTCs derived from five patients with ERα-positive breast cancer appeared more sensitive to TLK2 inhibition compared to those from four ERα-negative patients (Fig. 6g). Taken together, this observation suggests a discriminative anticancer effect of PTZs against high TLK2-expressing tumors. In addition to anti-HER2 therapies, these antipsychotic drugs can be repurposed as adjuncts for treating breast cancer patients with 17q-related chromothripsis.

## Discussion

The occurrence of chromothripsis, a single catastrophic event of chromosome shattering and reassembly, was detected in a broad range of tumor entities, including, but not limited to, hematopoietic malignancies, solid tumors, central nervous system tumors, and soft tissue tumors [16, 48–52]. This massive event of genomic scars may contribute to malignant transformation, in which end-joining-based repair can result in amplification of oncogenes, deletion of tumor suppressor genes, and formation of fusion genes [53, 54]. Although a number of studies have discussed the effects of chromothripsis in cancer development, few addressed the influence of transcription factors on the initiation of chromothriptic events [48, 53, 54]. We hypothesize that oncogenes are potential initiators of chromothripsis through frequent intra- and inter-chromosomal interactions with their target loci. Prolonged interactions enhance exchanges between two nonhomologous chromosomes through imprecise DNA repair, leading to widespread translocations and segmental duplications and deletions in or close to breakpoint junctions.

In the present study, we identified five genomic regions on 17q as potential hotspots of chromothripsis in breast cancer. Detailed genomic mapping further confirmed complex rearrangements between 20q13 and 17q23 that harbor a dense ERα hub and their corresponding target loci, respectively. Furthermore, 17q23 TADs may additionally interact with multiple ERα binding sites in other chromosome regions, leading to amplification observed in ~ 20% of breast cancer patients [55, 56]. This unique amplification event has also been reported in tumors of the brain [57, 58], lung [59, 60], liver [61, 62], pancreas [63], bladder [64] and testis [65], suggesting the important contribution of a target gene or genes located on 17q23 to the development of multiple tumor types.

One potentially druggable target gene closely linked to adverse clinical events in breast cancer patients is *TLK2* [43]. The gene encodes a nuclear serine/threonine kinase involved in the regulation of DDR signaling in response to DSBs [45]. Although the precise target for antitumor action remains to be defined, our siRNA knockdown analyses clearly indicate antineoplastic effects of TLK2 inhibition. This is in line with recent studies showing that ectopic expression of TLK2 results in enhanced aggressiveness [43]. We further evaluated the efficacy of pharmacological inhibition of TLK2 on breast cancer cell lines. While PTZs were originally developed to treat schizophrenia and related disease, some of the derivatives have also been shown to elicit antineoplastic effects in glioma [66, 67], leukemia [68, 69], melanoma [70], lung cancer [71], hepatocellular carcinoma [72], oral cancer [73], as well as in cancer stem cells [74]. Additionally, our in vitro observations suggest discriminatory antiproliferative and cytotoxic effects of PPH between high and low TLK2-expressing cancer cells. More importantly, the anticancer effect of PPH was validated ex vivo using patient-derived CTCs. Our observation can also be supported by a recent study that demonstrated a valid therapeutic effect of TLK2 inhibition in a preclinical model [43].

## Conclusions

This study demonstrates a new approach for identifying tumorigenic drivers from genomic regions highly susceptible to ERα-related chromothripsis. Specifically, we found the unique phenomenon of chromothripsis contributes to concordant upregulation of genes localized on 17q. Future interrogation of chromothriptic events that are associated with other oncogenic transcription factors could lead to the discovery of additional actionable targets. Our case study of the 17q23 region demonstrates that PPH exerts antineoplastic effects in breast cancer cells, at least in part, through the pharmacological targeting of TLK2-mediated DDR pathway. The present finding reinforces the idea that PTZ derivatives are potential candidates as repurposed therapeutics for treating breast cancer patients exhibiting 17q-related chromothripsis.

## Supplementary information

**Additional file 1: Figure S1.** The expression profile of breast tumors with PAM50 subtypes and normal controls of the 96 amplification-associated transcription coupling (ATC) loci from the five regions on 17q (A, 17q11.1-q11.2; B, 17q12-q21.2; C, 17q21.2-q21.31; D, 17q21.31-q21.33; E, 17q22-q24.1). The locations of the tumors of different subtypes are displayed by the solid lines with designated colors on the top of the heat map. **Figure S2**. Circos plots of inter- and intra- chromosomal rearrangements related to three 17q regions. **Figure S3.** Nanopore sequencing of inter-chromosomal rearrangements involving the 17q23 region. a. 17q23-associated rearrangements identified in MCF-7. Rearrangement frequencies were determined using previously generated whole-genome mate-pair sequencing data. b. Nanopore sequencing of the MCF-7 genome. A schematic flow chart (*left*) indicates the principle of Nanopore long-read sequencing. Unique molecular barcodes were incorporated into amplicons individually by PCR to enable multiplex sequencing of samples and the resultant reactions were then pooled. After end repair and A-tail reactions, leader and the hairpin adapters, each containing a motor protein (orange), were ligated to the end prepared DNA, followed by His-tag purification. On the MinION device, DNA molecules are pulled through a protein pore (gray) with motor proteins, producing 2D reads, which were consensus calls of the combined template and complement strands (red). PCR amplicons (*right*) spanning the 18 chromosomal breakpoints between chromosomes 17q23 and 20q13 were individually barcoded and pooled together for sequencing. The image of the desktop sequencing process on the MinION device (*left bottom*) was captured in our laboratory. c. Screen captures of representative NCBI-BLAST search outputs. DNA sequences from the 17q23 and 20q13 regions are shown in orange and blue, respectively.

**Additional file 2: Table S1.** Frequencies of dense-ERα-binding-site related chromosomal rearrangements in five targeted regions. **Table S2.** Primer sequences used in Nanopore sequencing. **Table S3.** Primer sequences used in CRISPR/Cas9 experiments. **Table S4.** Primer sequences used in copy-number analysis. **Table S5.** Demographic and clinical characteristics of CTC patient samples.

## Data Availability

1. Copy number and RNA-seq data of TCGA breast cancer cohort were from cBioPortal (http://download.cbioportal.org/brca_tcga.tar.gz) [75]. 2. Whole-genome DNA sequencing data of the TCGA-BRCA cohort were retrieved from GDC Data Portal (https://portal.gdc.cancer.gov/legacy-archive/search/f). 3. ChIP-seq data for ERα in MCF-7, T47D, and MDA-MB-134 cells were downloaded from Cistrome Data Browser (http://cistrome.org/db/#/) [20] from GEO accession number GSM2736194, GSM1908537 and GSM2931695, respectively. 4. The heatmap of Hi-C data of HMEC was generated in 3D Genome Browser [76] (http://promoter.bx.psu.edu/hi-c/view.php) with data from GEO accession number GSE63525. 5. The annotation of CTCF, H3K27me3, H3K36me3, H3K4me3, POLR2A and RNA-seq in Fig. 4a were visualized with data from GEO accession number GSM733724, GSM733722, GSM733707, GSM733712, GSM935456, and GSM325485, respectively.

## Abbreviations

ERα: Estrogen receptor α
ATC: Amplification-associated transcription coupling
TCGA: The Cancer Genome Atlas
TAD: Topologically associating domain
PTZ: Phenothiazine
PPH: Perphenazine
TFP: Trifluoperazine
TRD: Thioridazine
DDR: DNA damage response
CTC: Circulating tumor cell
