# Supplementary material for: ERα-related chromothripsis enhances concordant gene transcription on chromosome 17q11.1-q24.1 in luminal breast cancer
Source: BMC Med Genomics. 2020 May 14;13:69. doi: 10.1186/s12920-020-0729-7 (PMC7222439; doi:10.1186/s12920-020-0729-7)
Supplement: Supplementary file 1 — Additional file 1: Figure S1. The expression profile of breast tumors with PAM50 subtypes and normal controls of the 96 amplification-associated transcription coupling (ATC) loci from the five regions on 17q (A, 17q11.1-q11.2; B, 17q12-q21.2; C, 17q21.2-q21.31; D, 17q21.31-q21.33; E, 17q22-q24.1). The locations of the tumors of different subtypes are displayed by the solid lines with designated colors on the top of the heat map. Figure S2. Circos plots of inter- and intra- chromosomal rearrangements related to three 17q regions. Figure S3. Nanopore sequencing of inter-chromosomal rearrangements involving the 17q23 region. a. 17q23-associated rearrangements identified in MCF-7. Rearrangement frequencies were determined using previously generated whole-genome mate-pair sequencing data. b. Nanopore sequencing of the MCF-7 genome. A schematic flow chart (left) indicates the principle of Nanopore long-read sequencing. Unique molecular barcodes were incorporated into amplicons individually by PCR to enable multiplex sequencing of samples and the resultant reactions were then pooled. After end repair and A-tail reactions, leader and the hairpin adapters, each containing a motor protein (orange), were ligated to the end prepared DNA, followed by His-tag purification. On the MinION device, DNA molecules are pulled through a protein pore (gray) with motor proteins, producing 2D reads, which were consensus calls of the combined template and complement strands (red). PCR amplicons (right) spanning the 18 chromosomal breakpoints between chromosomes 17q23 and 20q13 were individually barcoded and pooled together for sequencing. The image of the desktop sequencing process on the MinION device (left bottom) was captured in our laboratory. c. Screen captures of representative NCBI-BLAST search outputs. DNA sequences from the 17q23 and 20q13 regions are shown in orange and blue, respectively. [file 12920_2020_729_MOESM1_ESM.pptx]

## Slide 1
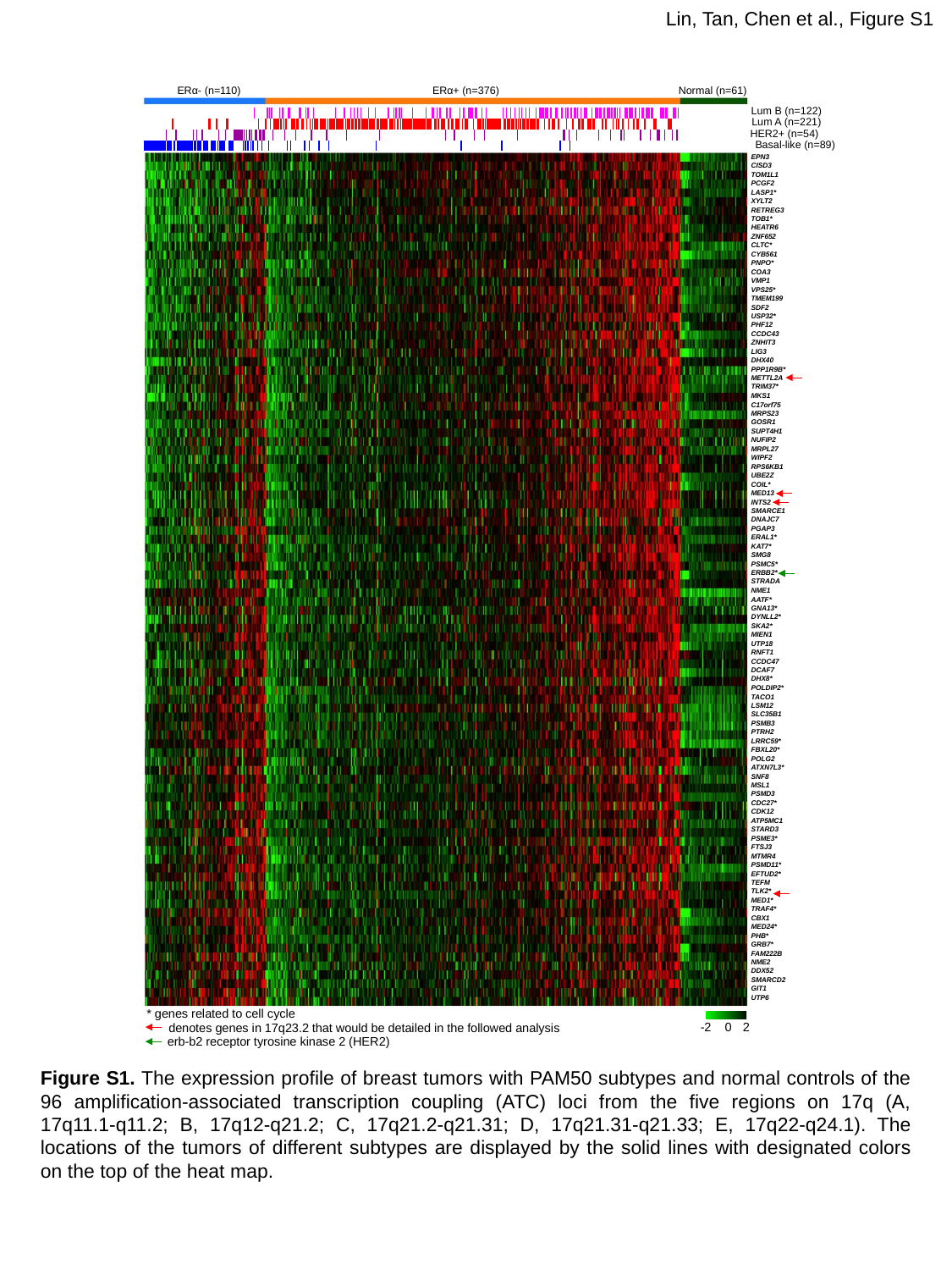

Lin, Tan, Chen et al., Figure S1
ERα- (n=110)
ERα+ (n=376)
Normal (n=61)
Lum B (n=122)
Lum A (n=221)
HER2+ (n=54)
Basal-like (n=89)
| EPN3 |
| --- |
| CISD3 |
| TOM1L1 |
| PCGF2 |
| LASP1\* |
| XYLT2 |
| RETREG3 |
| TOB1\* |
| HEATR6 |
| ZNF652 |
| CLTC\* |
| CYB561 |
| PNPO\* |
| COA3 |
| VMP1 |
| VPS25\* |
| TMEM199 |
| SDF2 |
| USP32\* |
| PHF12 |
| CCDC43 |
| ZNHIT3 |
| LIG3 |
| DHX40 |
| PPP1R9B\* |
| METTL2A |
| TRIM37\* |
| MKS1 |
| C17orf75 |
| MRPS23 |
| GOSR1 |
| SUPT4H1 |
| NUFIP2 |
| MRPL27 |
| WIPF2 |
| RPS6KB1 |
| UBE2Z |
| COIL\* |
| MED13 |
| INTS2 |
| SMARCE1 |
| DNAJC7 |
| PGAP3 |
| ERAL1\* |
| KAT7\* |
| SMG8 |
| PSMC5\* |
| ERBB2\* |
| STRADA |
| NME1 |
| AATF\* |
| GNA13\* |
| DYNLL2\* |
| SKA2\* |
| MIEN1 |
| UTP18 |
| RNFT1 |
| CCDC47 |
| DCAF7 |
| DHX8\* |
| POLDIP2\* |
| TACO1 |
| LSM12 |
| SLC35B1 |
| PSMB3 |
| PTRH2 |
| LRRC59\* |
| FBXL20\* |
| POLG2 |
| ATXN7L3\* |
| SNF8 |
| MSL1 |
| PSMD3 |
| CDC27\* |
| CDK12 |
| ATP5MC1 |
| STARD3 |
| PSME3\* |
| FTSJ3 |
| MTMR4 |
| PSMD11\* |
| EFTUD2\* |
| TEFM |
| TLK2\* |
| MED1\* |
| TRAF4\* |
| CBX1 |
| MED24\* |
| PHB\* |
| GRB7\* |
| FAM222B |
| NME2 |
| DDX52 |
| SMARCD2 |
| GIT1 |
| UTP6 |
* genes related to cell cycle
-2
0
2
denotes genes in 17q23.2 that would be detailed in the followed analysis
erb-b2 receptor tyrosine kinase 2 (HER2)
Figure S1. The expression profile of breast tumors with PAM50 subtypes and normal controls of the 96 amplification-associated transcription coupling (ATC) loci from the five regions on 17q (A, 17q11.1-q11.2; B, 17q12-q21.2; C, 17q21.2-q21.31; D, 17q21.31-q21.33; E, 17q22-q24.1). The locations of the tumors of different subtypes are displayed by the solid lines with designated colors on the top of the heat map.

## Slide 2
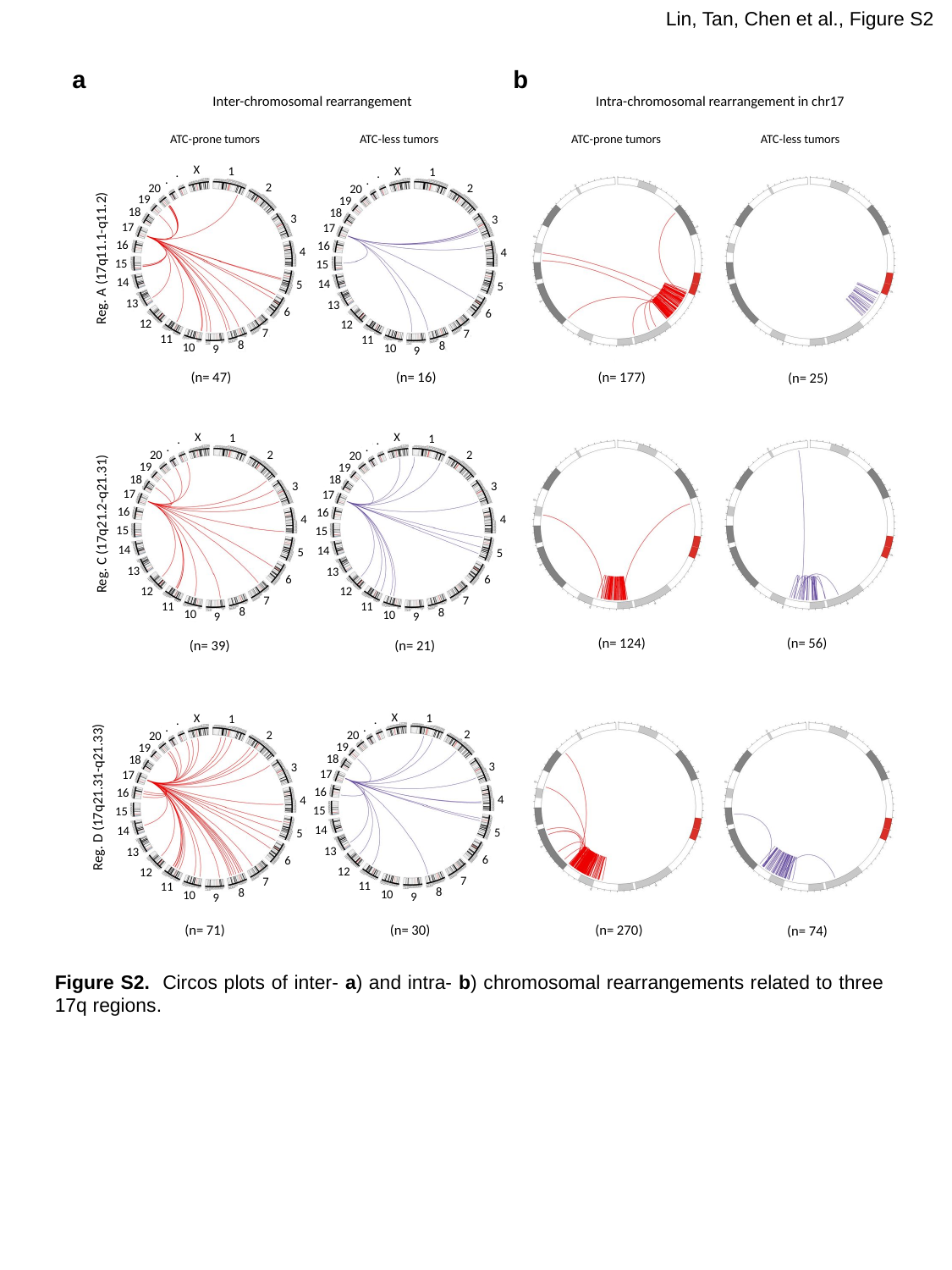

Lin, Tan, Chen et al., Figure S2
b
a
Intra-chromosomal rearrangement in chr17
Inter-chromosomal rearrangement
ATC-prone tumors
ATC-prone tumors
ATC-less tumors
ATC-less tumors
X
.
1
.
2
20
19
18
3
17
16
4
15
14
5
13
6
12
7
11
8
10
9
X
.
1
.
2
20
19
18
3
17
16
4
15
14
5
13
6
12
7
11
8
10
9
Reg. A (17q11.1-q11.2)
(n= 16)
(n= 47)
(n= 177)
(n= 25)
X
.
1
.
2
20
19
18
3
17
16
4
15
14
5
13
6
12
7
11
8
10
9
X
.
1
.
2
20
19
18
3
17
16
4
15
14
5
13
6
12
7
11
8
10
9
Reg. C (17q21.2-q21.31)
(n= 124)
(n= 56)
(n= 21)
(n= 39)
X
.
1
.
2
20
19
18
3
17
16
4
15
14
5
13
6
12
7
11
8
10
9
X
.
1
.
2
20
19
18
3
17
16
4
15
14
5
13
6
12
7
11
8
10
9
Reg. D (17q21.31-q21.33)
(n= 30)
(n= 270)
(n= 71)
(n= 74)
Figure S2. Circos plots of inter- a) and intra- b) chromosomal rearrangements related to three 17q regions.

## Slide 3
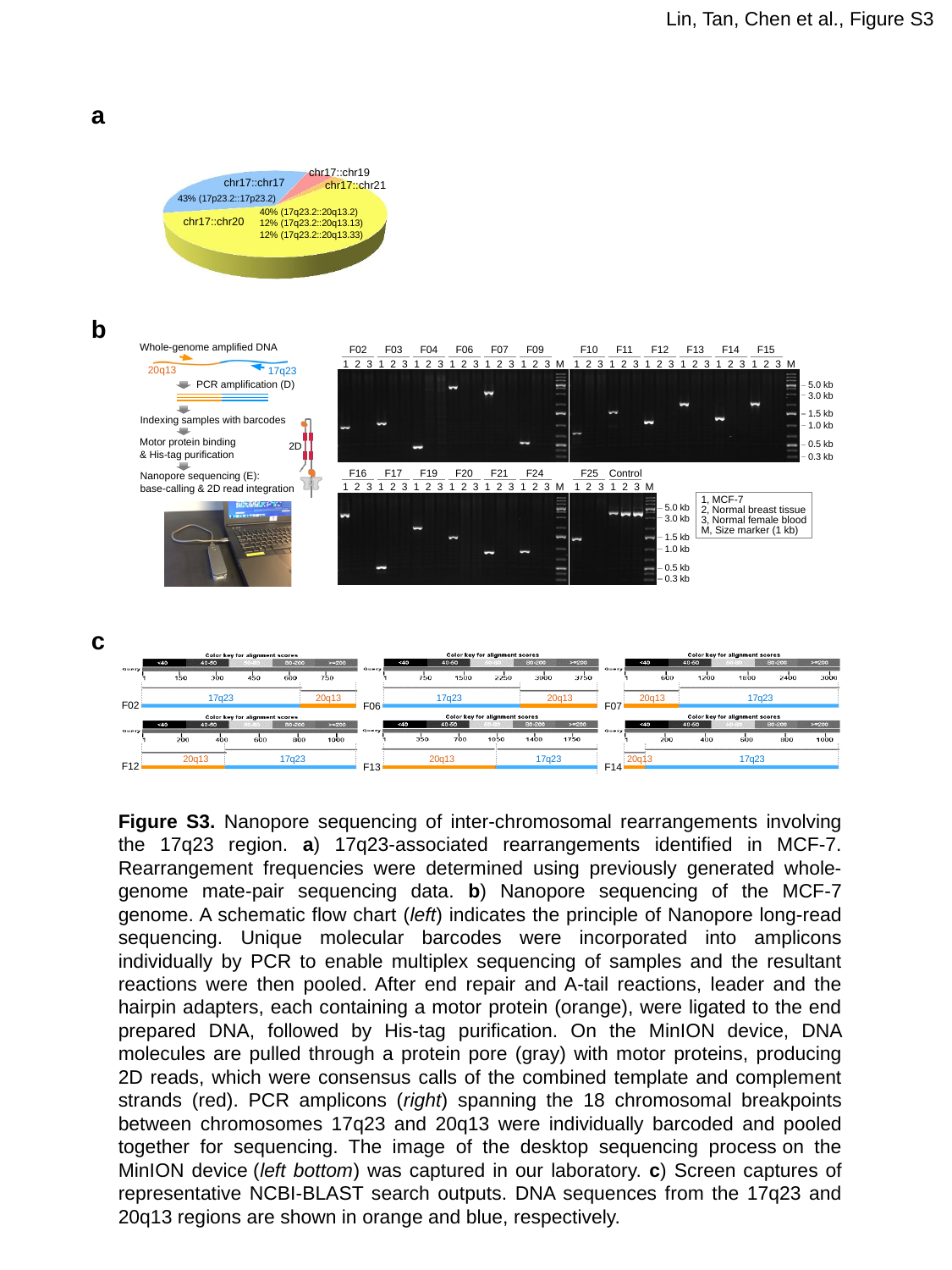

Lin, Tan, Chen et al., Figure S3
a
chr17::chr19
chr17::chr17
chr17::chr21
43% (17p23.2::17p23.2)
40% (17q23.2::20q13.2)
12% (17q23.2::20q13.13)
12% (17q23.2::20q13.33)
chr17::chr20
b
Whole-genome amplified DNA
20q13
17q23
PCR amplification (D)
Indexing samples with barcodes
Motor protein binding
& His-tag purification
2D
Nanopore sequencing (E):
base-calling & 2D read integration
F02
F03
F04
F06
F07
F09
F10
F11
F12
F13
F14
F15
1
2
3
1
2
3
1
2
3
1
2
3
1
2
3
1
2
3
M
1
2
3
1
2
3
1
2
3
1
2
3
1
2
3
1
2
3
M
5.0 kb
3.0 kb
1.5 kb
1.0 kb
0.5 kb
0.3 kb
F16
F17
F19
F20
F21
F24
F25
Control
1
2
3
1
2
3
1
2
3
1
2
3
1
2
3
1
2
3
M
1
2
3
1
2
3
M
1, MCF-7
2, Normal breast tissue
3, Normal female blood
M, Size marker (1 kb)
5.0 kb
3.0 kb
1.5 kb
1.0 kb
0.5 kb
0.3 kb
c
17q23
20q13
17q23
20q13
20q13
17q23
F02
F06
F07
20q13
17q23
20q13
17q23
20q13
17q23
F12
F14
F13
Figure S3. Nanopore sequencing of inter-chromosomal rearrangements involving the 17q23 region. a) 17q23-associated rearrangements identified in MCF-7. Rearrangement frequencies were determined using previously generated whole-genome mate-pair sequencing data. b) Nanopore sequencing of the MCF-7 genome. A schematic flow chart (left) indicates the principle of Nanopore long-read sequencing. Unique molecular barcodes were incorporated into amplicons individually by PCR to enable multiplex sequencing of samples and the resultant reactions were then pooled. After end repair and A-tail reactions, leader and the hairpin adapters, each containing a motor protein (orange), were ligated to the end prepared DNA, followed by His-tag purification. On the MinION device, DNA molecules are pulled through a protein pore (gray) with motor proteins, producing 2D reads, which were consensus calls of the combined template and complement strands (red). PCR amplicons (right) spanning the 18 chromosomal breakpoints between chromosomes 17q23 and 20q13 were individually barcoded and pooled together for sequencing. The image of the desktop sequencing process on the MinION device (left bottom) was captured in our laboratory. c) Screen captures of representative NCBI-BLAST search outputs. DNA sequences from the 17q23 and 20q13 regions are shown in orange and blue, respectively.
